# Stimuli‐responsive silk fibroin for on‐demand drug delivery

**DOI:** 10.1002/SMMD.20220019

**Published:** 2023-02-16

**Authors:** Xiang Lin, Lijun Cai, Xinyue Cao, Yuanjin Zhao

**Affiliations:** ^1^ Department of Rheumatology and Immunology Nanjing Drum Tower Hospital School of Biological Science and Medical Engineering, Southeast University Nanjing China; ^2^ Oujiang Laboratory (Zhejiang Lab for Regenerative Medicine, Vision and Brain Health) Wenzhou Institute University of Chinese Academy of Sciences Wenzhou China

**Keywords:** biomedical, hydrogel, on‐demand drug delivery, silk fibroin, stimuli‐responsive

## Abstract

Stimuli‐responsive “smart” hydrogel biomaterials have attracted great attention in the biomedical field, especially in designing novel on‐demand drug delivery systems. As a handful natural biomaterial approved by US Food and Drug Administration, silk fibroin (SF) has unique high temperature resistance as well as tunable structural composition. These properties make it one of the most ideal candidates for on‐demand drug delivery. Meanwhile, recent advances in polymer modification and nanomaterials have fostered the development of various stimuli‐responsive delivery systems. Here, we first review the recent advance in designing responsive SF‐based delivery systems in different stimulus sources. These systems are able to release mediators in a desired manner in response to specific stimuli in active or passive manners. We then describe applications of these specially designed responsive delivery systems in wound healing, tumor therapy, as well as immunomodulation. We also discuss the future challenges and prospects of stimuli‐responsive SF‐based delivery systems.

## Graphical abstract

Responsive SF‐based materials need to respond to both exogenous (acoustic, electric, magnetic and electromagnetic radiation, etc.) and endogenous stimuli (pH responsiveness, enzyme responsiveness) to trigger on‐demand release processes. These systems are able to release mediators in a desired manner in response to specific stimuli in active or passive manners. The reponsive delivery system can be employed in different therapeuric applications.

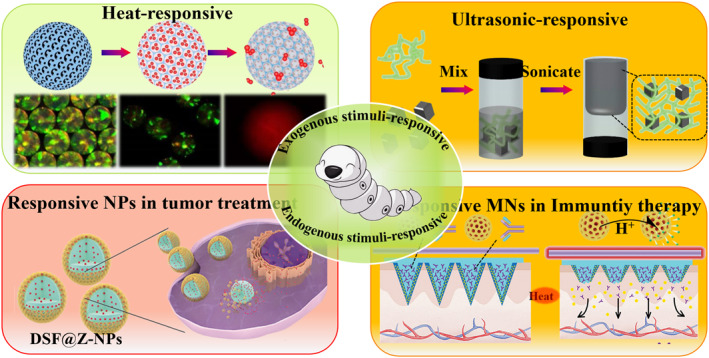

1

**Key points**

- Review on the recent advance in design of silk fibroin based stimuli‐responsive systems
- Therapeutic applications of stimuli‐responsive silk fibroin are discussed
- More safety silk fibroin delivery systems remain many challenges

## 2 INTRODUCTION

Last decades have witnessed an unprecedented revolution in drug delivery. Intensive long‐lasting controlled release systems were developed for chronic diseases, which require long‐lasting drug delivery, or targeted drug delivery followed by targeted clearance of tumor tissues.^1^ However, most of these approaches rely on the degradation of polymers as well as the physicochemical conditions in target tissues, leading to poor control of release dose, times, and site of action.^2^ To address these problems, scientific attention has been devoted to develop on‐demand delivery based on stimuli‐responsive drug delivery systems. The on‐demand delivery can realize controllable release of drugs to specific sites, which offers considerable advantages over passive delivery systems, including improved bioavailability, complex release profiles, and reduced deleterious side effects.^3^ Aiming at better control, many responsive materials were introduced for the improvement of on‐demand drug delivery. Among them, responsive silk fibroin (SF)‐based delivery system is one of the most promising approaches. As a handful Food and Drug Administration (FDA)‐approved natural biomedical material, SF is a natural polymeric fibrous protein derived from silkworm, which has excellent biocompatibility, low immunogenicity, as well as feasibility of modification.^4^ The SF‐based stimuli‐responsive materials showed great potential in controlled drug delivery systems, from sustained release of drugs to the construction of chemotherapy platforms with targeted therapeutic effects, which serve as ideal therapeutic tools for drug delivery, regenerative medicine, and so on.

To design on‐demand delivery systems, responsive SF‐based materials need to respond to both exogenous (acoustic, electric, magnetic and electromagnetic radiation, etc.) and endogenous stimuli (pH responsiveness and enzyme responsiveness) to trigger on‐demand release processes.^5^ SF‐based delivery systems with different stimulus responsiveness can be generated by changing the composition and crystallinity of the SF material.^6^ These approaches have led to the emergence of a wide variety of stimulus‐responsive SF delivery carriers with different structures, components, and multiple functions.^7^ Herein, a review on stimuli‐responsive SF applied for on‐demand drug delivery is presented. Firstly, we examine recent advances about different stimulus sources for SF‐based delivery systems. These systems are able to respond to specific stimuli in active or passive manners to control the biodistribution of drugs. We then describe applications of these specifically designed responsive delivery systems for trauma repair, tumor therapy, and immunomodulation. Finally, we provide the future challenges for SF‐based responsive delivery systems. This review is anticipated to help readers to stay abreast of the research frontiers in this field.

## 3 DESIGN OF SF‐BASED STIMULI‐RESPONSIVE SYSTEMS

The specificity of the stimulus response and the response to endogenous cues inherent in the tissue microenvironment offers the possibility to develop new on‐demand drug delivery strategies.^8^ Notably, in order to construct more SF based on‐demand delivery carriers with exogenous responsive properties, magnetically responsive nanoparticles, thermally responsive polymers and specific materials with photothermal effects have been integrated into SF‐based drug delivery systems for different biomedical scenarios. These approaches have led to the emergence of various stimulus‐responsive SF delivery carriers with different structures, components, and multiple functions.^9^ In this chapter, we focus on the construction and functional characterization of different SF‐based delivery systems, mainly including various exogenous and endogenous stimuli including temperature, light, magnetic field, pH, and enzyme responses.

### 3.1 Exogenous stimuli‐responsive

#### 3.1.1 Thermal responsive

SF has excellent thermal stability.^10^ The traditional alkaline extraction process of regenerated silk proteins can be carried out at temperatures up to 100°C without affecting their protein structures. Thus, taking advantage of its thermal stability, controllable thermo‐responsive drug release can be achieved by integrating with other materials with thermosensitive properties.^11^ For instance, the hybrid gelatin/SF hydrogels are expected to constitute attractive biomaterials due to their customizable composition and temperature‐dependent swelling and release properties.^12^ Common gelatin solutions of animal origins typically convert from random helices to triple helix macromolecules at room temperature (below 30°C), resulting in physical cross‐linking. This cross‐linked network is reversible with increasing temperature. In contrary to gelatin, SF introduced *β*‐sheet in SF through exposure to methanol or methanol/water solution to promote *β*‐sheet formation in the crystalline region of SF, thereby helping to stabilize the hydrogel at high temperature.^12^ Swelling kinetics confirmed that the gelatin/SF hydrogel showed little mass loss at 20°C, but sustained protein loss at 37°C, due to the slow release of gelatin molecules into the surrounding aqueous solution.

**FIGURE 1:**
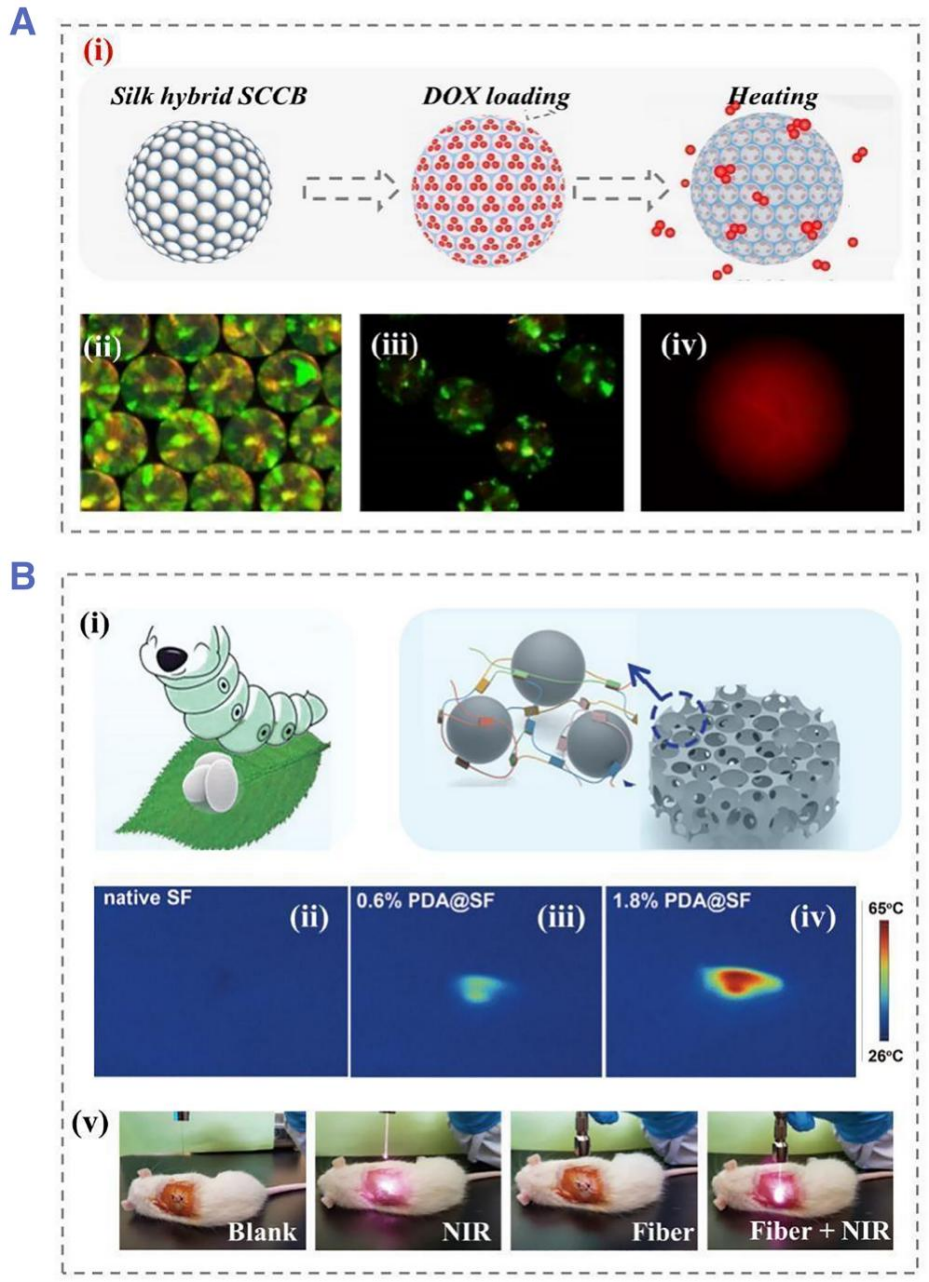
(A) The DOX loading and release process of silk hybrid scaffold, (i) generation procedure of SF scaffold with an inverse opal structure, (ii) the scaffold fabricated by nanoparticles (green) with diameters of 260 nm, (iii) PAIPAM filled particles, (iv) the fluorescent images of DOX release after seven cycles. Reproduced with permission.^14^ Copyright 2020, Elsevier. (B) NIR responsive process of a photo‐responsive silk nanoparticles: (i) Functionalized SF‐based PDA NPs, (ii) temperature images of the native SF, (iii) 0.6% PDA@SF and the (iv) 1.8% PDA@SF, and (v) illustration of the photothermal effect of 1.2% PDA@SF scaffold irradiated in different groups. Reproduced with permission.^17^ Copyright 2021, John Wiley and Sons. DOX, doxorubicin; NIR, near‐infrared; NP, nanoparticles; PDA, polydopamine; SF, silk fibroin.

Besides, poly(N‐isopropylacrylamide) (PNIPAM) has both hydrophilic amido groups and hydrophobic isopropyl groups, making both the aqueous solution of linear PNIPAM and the cross‐linked PNIPAM hydrogel exhibit temperature‐sensitive properties.^13^ Combining heat responsive PNIPAM with SF‐based inverse opal scaffolds could form thermo‐responsive microcarriers for sustained‐drug release. In detail, PNIPAM hydrogels with therapeutic drugs were fulfilled into inverse opal scaffolds benefiting from the homogeneous porous microstructure and interconnected nanopores of SF‐based scaffolds after several cycles of warming and cooling processes; the drug was released in a controlled manner into the simulated humoral environment (Figure 1A).^14^ In addition, due to its excellent biocompatibility and the high specific surface areas, the SF hydrogel also supported the adhesion and growth of normal cells, which contributed to the regeneration of diseased tissue.

#### 3.1.2 Light responsive

Due to their non‐invasive and wireless control possibilities, various light‐responsive systems have been designed over the past few decades.^15^ These photo‐responsive carriers can utilize specific wavelengths of light in different wavelength (ultraviolet and near‐infrared) regions to achieve control of the release manner.^16^ In this case, commonly used on‐demand delivery is initiated by the modification of photosensitivity‐induced nanocarrier structures. For example, polydopamine (PDA), the main pigment of natural melanin, could absorb light wavelengths in the near‐infrared region.^17^ The synthesized PDA nanoparticles (NPs) had rough surface and porous structure. The size of PDA NPs could be easily controlled by adjusting the molar ratio of SF to PDA. The introduction of the synthesized PDA nanoparticles into the silk‐cellulose porous scaffold led to a photo‐responsive composite microcarriers (Figure 1B). The temperature was increased under near‐infrared (NIR) irradiation in a controlled manner, which could achieve strong photothermal cytotoxicity to cancer cells. In addition to PDA,^18^ black phosphorus (BP) quantum dots could also be employed for photo‐responsive hydrogels. Some studies have integrated BP with SF to prepare on‐demand drug release microcarriers, where the SF was used as novel and robust therapeutic systems that exhibit a photothermal effect on tumor cells.^18^ In detail, the integration of BP quantum dot NPs with SF hydrogel scaffolds can be achieved through oil‐in‐water emulsions. The addition of BP quantum dots enables energy transfer by thermal stimulation, and in this way, osteoclast differentiation could be inhibited.

#### 3.1.3 Electromagnetic response

**FIGURE 2:**
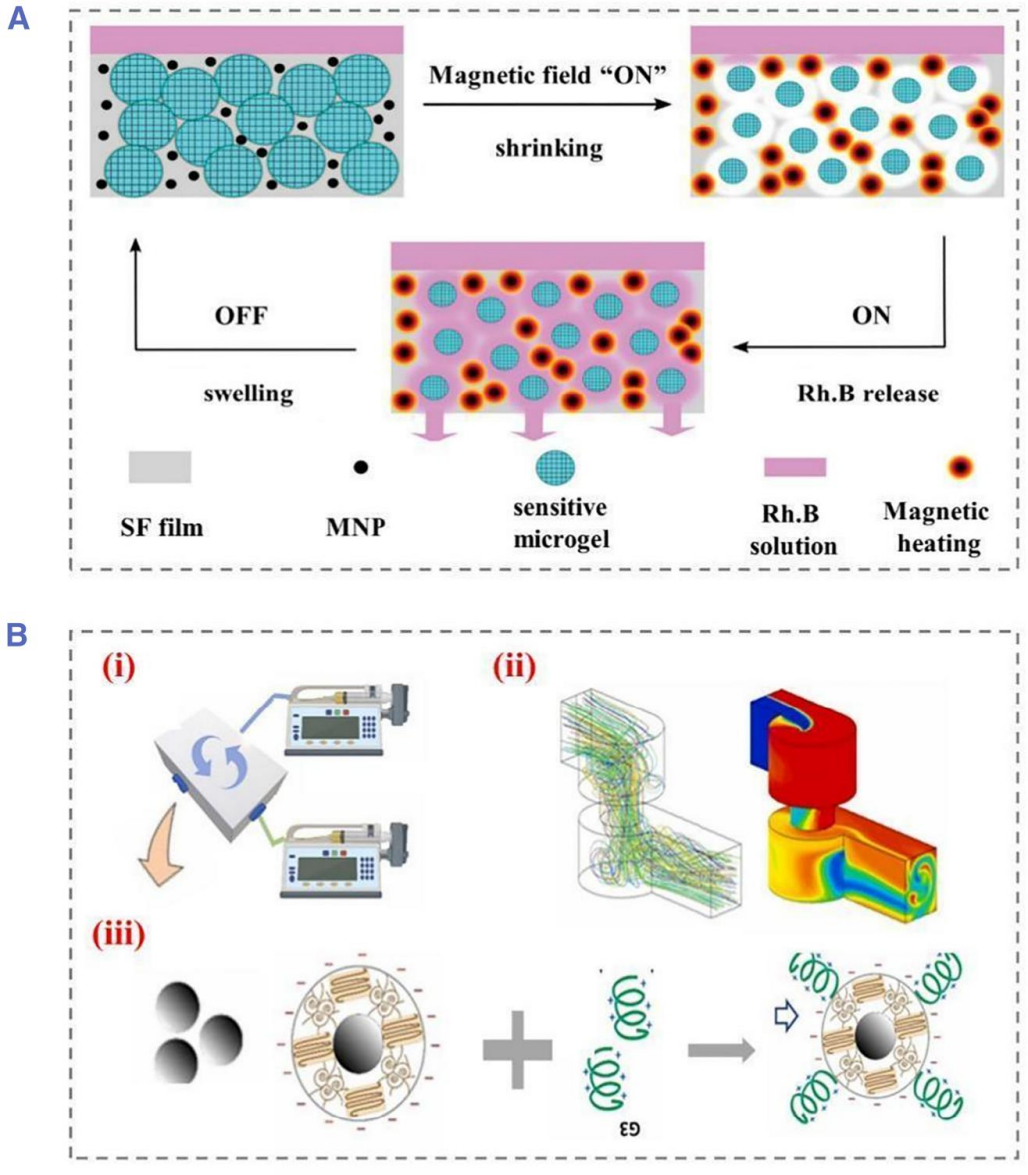
(A) Silk membranes with magnetic nanoparticles and heat/pH‐sensitive microgels for drug delivery controlled by exogenous magnetic fields, Rhodamine B (Rh. B) fluorescent dye was used as a model drug to demonstrate its working principle. Reproduced with permission.^21^ Copyright 2020, John Wiley and Sons. (B) A microfluidic platform to generate magnetic silk nanoparticles (MSNPs) followed by G3 functionalized preparation, (i) schematic diagram of microfluidic generation, (ii) liquid flow inside the device, and (iii) the binding process of anticancer peptide G3. Reproduced with permission.^22^ Copyright 2022, Elsevier.

Magnetically responsive nanomaterials, especially magnetic nanoparticles (MNPs), possess numerous properties like porous structure, low intraparticle diffusivity, superparamagnetic properties, etc.^19^ In addition, the features of non‐contact control and non‐invasive injury ensure safety therapeutic effects in clinic. Basically, SF is non‐conductive and needs to be combined with conductive hydrogels or MNPs to form composites.^20^ For example, MNPs were embedded in SF to generate composite membranes. The SF served as a main substance and the permeability was prepared by MNPs (localized heating source). The on/off switching of an alternating magnetic field generated heat, which caused a reversible change in the volume of the hydrogel network due to the shrinkage/swelling of the hydrogels (Figure 2A).^21^ The drug release process simulated by rhodamine B was achieved by adjusting the content of SF hydrogels and MNPs as well as the thickness of the film. In another attempts, magnetic silk nanoparticles in size ranging from 129 to 232 nm were directly produced by a microfluidic device. By adjusting the solvent ratio and liquid flow rate, moisture can be removed from the silk structure, which enabled size control over the formation of SF nanoparticles. This novel microfluidic device with a mixer enabled the preparation of silk‐based MNPs with a desirable diameter (Figure 2B).^22^ Furthermore, the surface of MNPs could be functionalized with various small molecular peptides, such as anticancer peptide G(IIKK)3I‐NH_2_ (G3), to promote the treatment effect.

#### 3.1.4 Ultrasonic response

**FIGURE 3:**
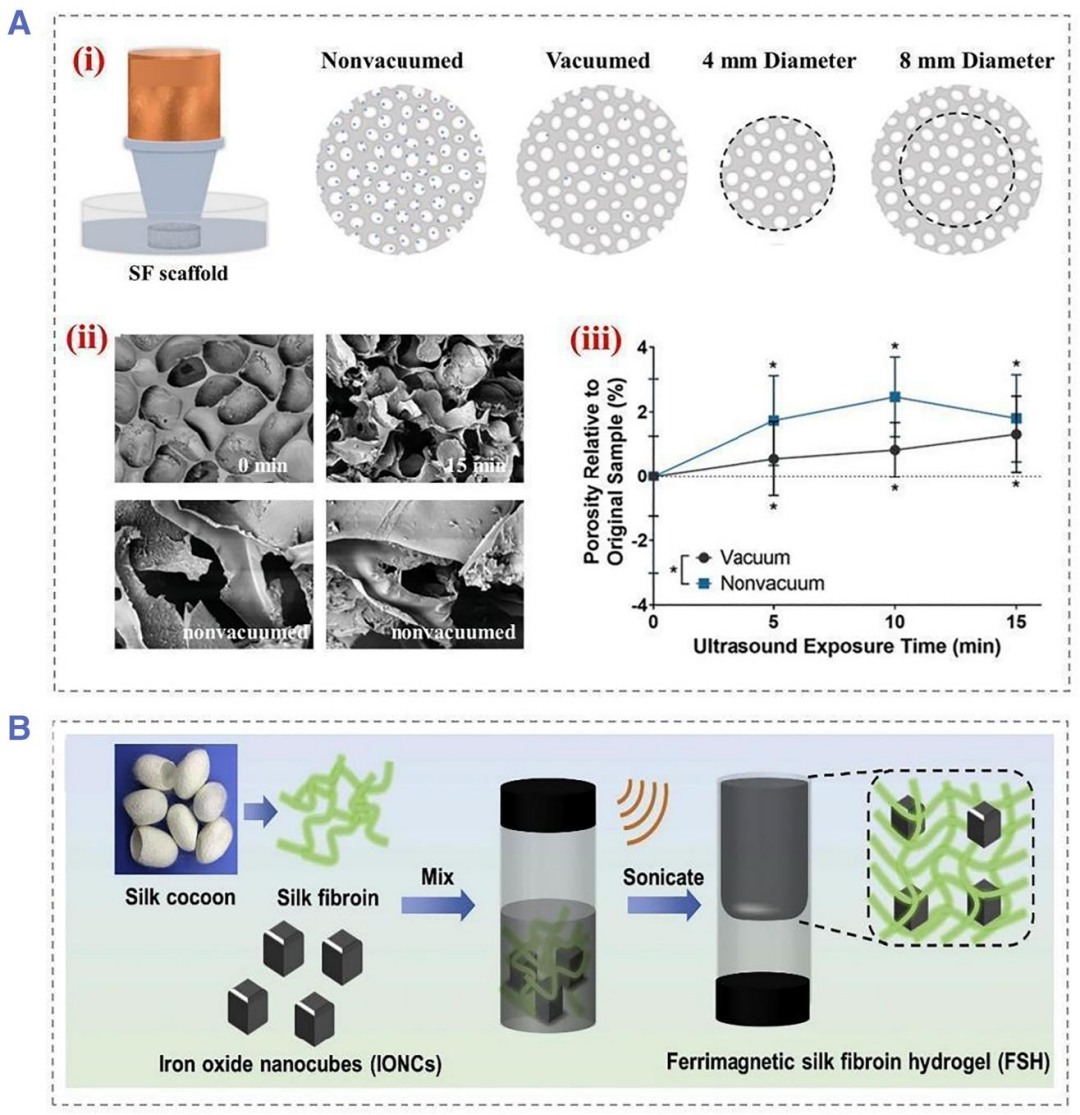
(A) Schematic images showed the sonication setup for the initial experiment. (i) The collimator is filled with ultrasonic transmission gel to ensure that the ultrasonic waves reach the silk scaffold. The microbubbles are represented by blue dots, while the white ovals represent the pores present in the silk scaffold. (ii) SEM images of vacuum exposure for 0 and 15 min, respectively. SEM images showing significant changes to the surface after sonication, as well as changes in the surface pore walls after exposure to non‐vacuum. (iii) Statistical graph of porosity showed significant increases for each sonication time period as well as vacuum versus non‐vacuum samples. Reproduced with permission.^24^ Copyright 2021, John Wiley and Sons. (B) Schematic diagram of preparation of ferromagnetic silk fibroin hydrogel. Reproduced with permission.^25^ Copyright 2020, Elsevier. SEM, scanning electron microscope.

Ultrasound has been used extensively for medical purpose, such as ultrasound imaging and in vivo calculi therapy. Due to its depth and concentrated penetration, ultrasound facilitates the wireless on‐demand drug release process in targeted areas.^23^ The functional impact of ultrasound on tissue has been intensively studied. In general, ultrasound mainly achieves drug control and treatment through temperature change, cavitation effect, and polymer degradation. Different types of ultrasounds have different degrees of tissue damage. To develop biomaterial systems with adjustable degradability after implantation, therapeutic ultrasound can be used as a promoting way to alter the distribution of filamentous protein degradation. Unlike conventionally applied ultrasound, clinical therapeutic ultrasound has a higher intensity and lower frequency. In fact, low‐intensity focused ultrasound (LIFU) could be used to induce therapeutic interactions for clinical use (FDA‐approved) (Figure 3A).^24^ Research studies have reported the controlled degradation of LIFU‐based SF scaffold in a passive manner and have found that acoustic cavitation was the main mechanism for altering the interpretation curve. This means that therapeutic ultrasound can also be used to improve drug uptake in cells and tissues.

In contrast to photo‐thermal therapy, the MNPs‐mediated ultrasound therapy is not depth‐limited. For example, ultrasound‐activated thermotherapy was performed by confining hydrophilic iron oxide nanocubes (IONCs) within the polymer matrix of injectable SF hydrogels. By applying an alternating magnetic field, tumors are ablated without depth restriction by converting electromagnetic energy into thermal energy in an alternating magnetic field (Figure 3B).^25^ In addition, ultrasound has the ability to strongly penetrate as well. SF nanoparticles containing FITC‐BSA are able to efficiently penetrate the iris via the transscleral route under ultrasound without damaging the ocular tissue or the particles themselves. Furthermore, the researchers investigated the triggered release behavior of SF hydrogels through the development of experimental and computational models. These stimuli are triggered at specific time intervals using ultrasound. Different drug release rates can be determined using different power intensities and induction times. Computer simulations show that ultrasound can significantly enhance drug release rates by as much as 10‐fold over the release rates without ultrasound stimulation. Therefore, on‐demand drug delivery can be achieved by adjusting the ultrasound power and induction time.

### 3.2 Endogenous stimuli‐responsive

#### 3.2.1 pH‐sensitive responsive

There are many applications for pH‐responsive materials since they are able to expand and contract reversibly in different pH solutions, such as drug delivery systems and chemical sensors.^26^ The proportion of the secondary structure in SF materials plays an important role in biological properties. SF contains several different secondary structures, such as *α*‐helices (Silk I), *β*‐sheet (Silk II), and amorphous regions.^27^ SF‐based coatings, however, contain either a high content of *β*‐sheets or *α*‐helices, depending on their equilibrium state. A high degree of orientation among the crystalline sheets of the SF‐based coatings usually modulates degradation kinetics, substrate stiffness, and biocompatibility. In response to external conditions such as pH, the secondary structure and conformation of SF can be flexibly altered. Low pH can enhance interchain aggregation due to highly hydrophilic groups, whereas at high pH, the conformation is stretched because of repulsion of charged carboxyl groups.^28^ Furthermore, it has been demonstrated that the initial burst release behavior of *β*‐sheet structures is significantly greater than that of *α*‐helices.

**FIGURE 4:**
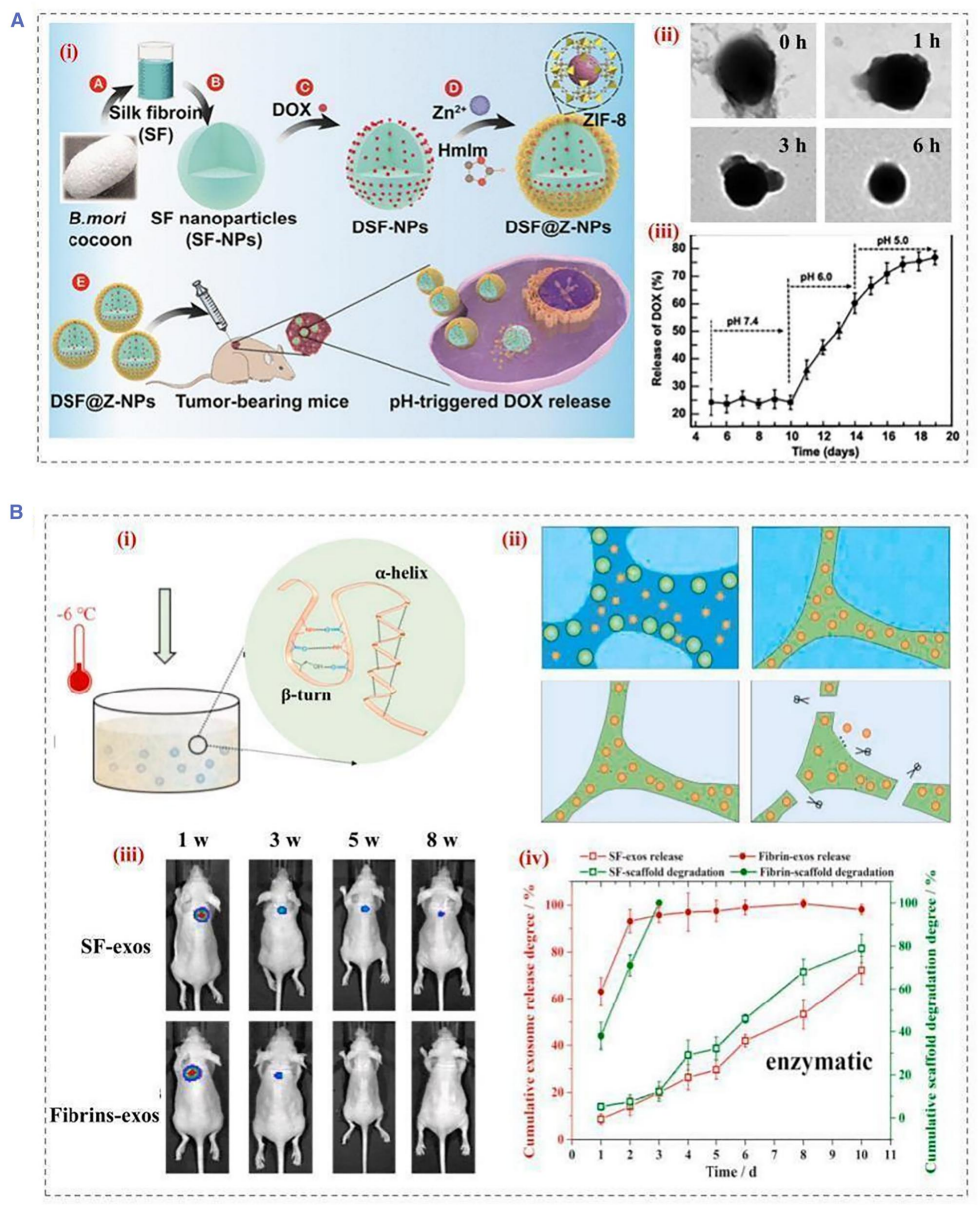
(A) Schematic illustration of (i) the development of DSF@Z‐NPs and the selective release of DOX from DSF@Z‐NPs into cancer cells, (ii) the release process of NPs in PBS buffers with pH = 5.0 in different times, (iii) DOX release process in a controlled manner. Reproduced with permission.^29^ Copyright 2021, American Chemical Society. (B) Schematic illustration of (i) the cryo self‐assembly process, (ii) mechanisms for sustained exosome release dominated by degradation, (iii) in vivo distribution of scaffolds carrying exosomes within 8 weeks, and (iv) exosome release and scaffold degradation profiles under enzymatic conditions. Reproduced with permission.^31^ Copyright 2022, The Authors, published by Elsevier. DOX, doxorubicin; NP, nanoparticles; PBS, phosphate buffered saline.

SF chains are negatively charged due to the presence of acidic aspartate and glutamate residues, limiting their pH response. The metal‐organic framework (MOF) is sensitive to acidic environments. It can be used for gas separation, storage, catalysis, and enzymatic reactions. To make SF more pH responsive, Chen et al. employed SF and MOF to develop new drug carriers to release drugs selectively in cancer cells' acidic intracellular environment (Figure 4A).^29^ Besides, the pH‐responsive cationic hydrogels could also formed by grafting *ε*‐Poly‐(L‐lysine) (ε‐PLL) with silk proteins and cross‐linking with Horseradish Peroxidase/H_2_O_2_.^27^ ^b^ The use of cationic serine proteins in skin scaffolds and gene delivery had been demonstrated by using a cationic peptide to modify SF‐based hydrogels with very low swelling of pure SF hydrogels. In contrast, SF hydrogels with different *ε*‐PLL grafting rates are sensitive to acidic pH and have a high degree of swelling at low pH values.

#### 3.2.2 Enzyme responsive

Analysis of the expression profiles of enzymes (such as proteases, phospholipases, and glycosidases) observed in pathological conditions, such as cancer or inflammation, can identify means of on‐demand drug delivery for therapeutic intervention.^15^, ^30^ In most enzyme‐mediated drug delivery systems, enzymes are present in the extracellular environment. Recently, a cryo‐sponge based on the SF protein was able to perform exocytosis release in a controlled manner (Figure 4B).^31^ The release of exosomes from the silk scaffold was primarily an enzymatic degradation process. An amorphous state of the protein chains can be transformed into Silk I structure by controlling the reaction temperature below the glass transition temperature of the SF. Silk scaffolds were used to encapsulate the exosomes during cryo‐self‐assembly. In another attempt, lysosomal proteases were able to reverse multidrug resistance by deconstructing silk protein hydrogels, leading to an abrupt release of doxorubicin (DOX) into the nucleus for inducing effective cell death.

Despite their ability to in situ eliminate tumor cells, reactive oxygen species (ROS) are constantly influenced by the tumor microenvironment (TME). To solve this problem, the SF delivery system can be employed to generate nano enzymes with tumor microenvironmental responsiveness. For example, one‐step reduction was used to synthesize AuPt@SF (APS) bimetallic nano enzymes using SF as a mineralization inducer and a sacrificial template.^32^ Such nano‐enzymes could enhance nano‐catalytic therapy by using intrinsic TME wires to overcome their limitations. Glucose oxidation can effectively elevate intracellular H_2_O_2_ levels in the presence of APS. APS nano‐enzymes could generate radicals such as superoxide and hydroxyl from adsorbed oxygen and endogenous H_2_O_2_ due to their mimetic activity and mimicry of oxidases and peroxidases. The generated ROS were further protected by depleting reduced glutathione to glutathione disulfide for enhancing therapeutic effects. This enzymatic reaction consumes glucose and enhances the production of ROS, effectively destroying tumor cells through detrimental tumor starvation and irreversible oxidative stress damage.

## 4 THERAPEUTIC APPLICATIONS OF STIMULI‐RESPONSIVE SILK FIBROIN

The delivery of drugs and genes needs to arrive at the desired location at the desired point in time, crossing barriers such as biological barriers, enzymatic or hydrolytic degradation, and solubility. The introduction of responsive SF brings many new implementations, which brings enhanced performance of SF‐based biomaterials. Therefore, drug delivery with multiple stimuli‐responsive carriers may provide new therapeutic options, and ultrasound, in designing smart materials for on‐demand delivery. In this section, we reviewed applications for wound healing, tumor therapy, immunotherapy, and other SF‐based responsive biomaterials.

### 4.1 Wound healing

**FIGURE 5:**
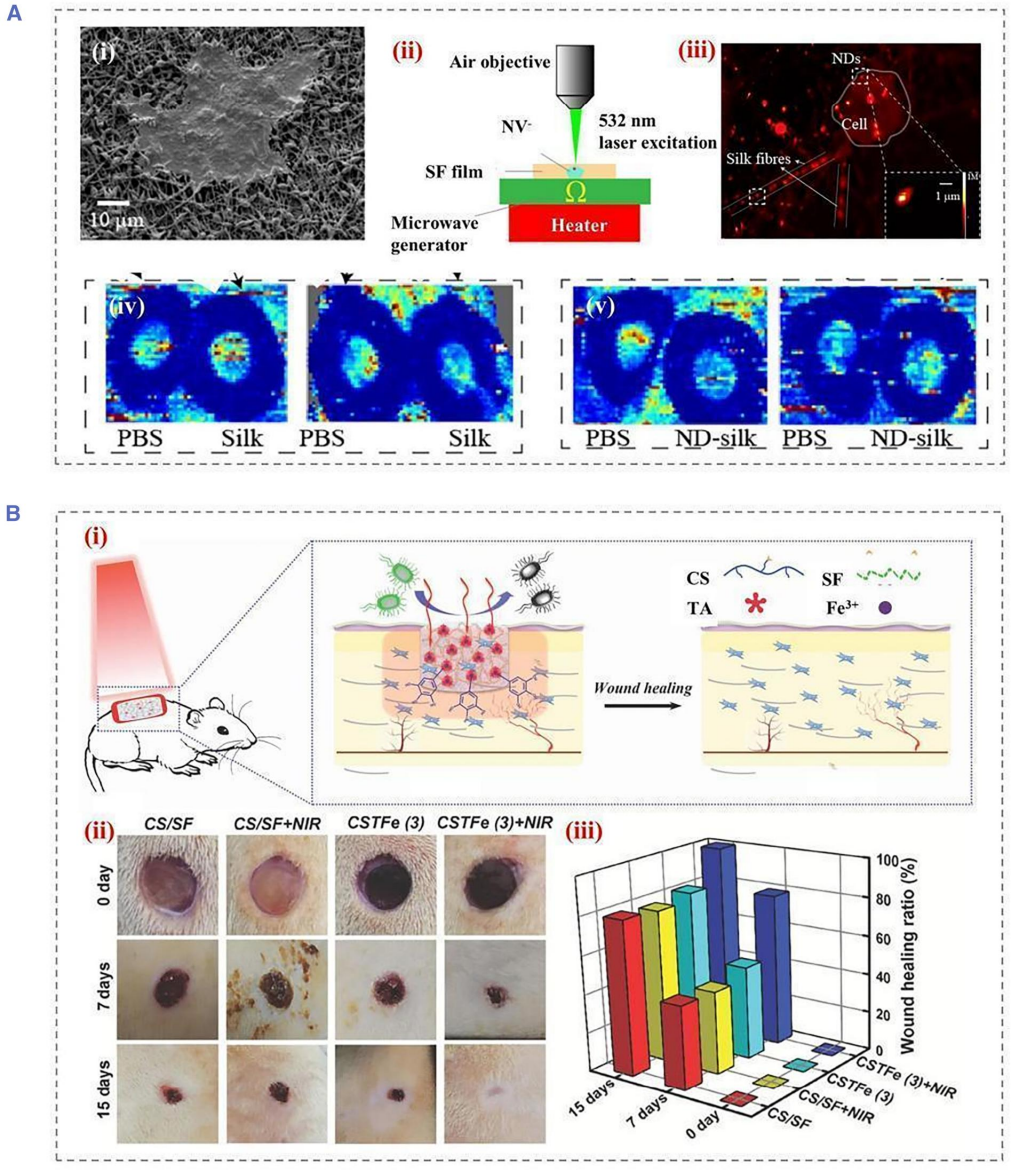
(A) SEM images of (i) HaCaT cells grown on ND‐silk fiber membranes, (ii) schematic diagram of the setup for photoexcitation and temperature detection of NDs at NV^−^ centers, (iii) 100 × 100 μm^2^ fluorescence image of ND^−^ silk membrane in cell culture. The dashed box shows two representative NDs, while the inset within the solid outline box shows an enlarged region of the selected region of interest. Wound blood perfusion was imaged on day 3 (iv) and day 7, (v) post‐injury in SF membrane‐treated mice using laser Doppler. Reproduced with permission.^34^ Copyright 2020, American Chemical Society. (B) Schematic diagram of (i) preparation of cryogel and application of wound repair, (ii) images of the wounds at day 0, 7, and 15, respectively, (iii) the wound healing ratio with various treatments at the different healing period. Reproduced with permission.^35^ Copyright 2019, John Wiley and Sons. SEM, scanning electron microscope; SF, silk fibroin.

Wound healing is a complex process that involves a number of steps mainly involving hemostasis, inflammatory, proliferative, and maturation process. Infections caused by bacteria often cause severe wound inflammation and death.^33^ Consequently, effective and rapid wound healing strategies are needed for treating skin injuries.^33^ ^a,^ ^33^ ^b^ Photothermal treatment (PPT) has gained much attention due to its remote control, deep tissue penetration, and accelerated tissue regeneration (Figure 5A).^34^ Due to its different antibacterial mechanism from antibiotics, PPT is effective in avoiding the development of drug resistance. Attracted by this, Yu et al. proposed a multifunctional SF composite wound dressing by mixing the natural product chitosan and SF followed by freeze‐drying. The composites made from chitosan and SF are used for wound dressing platforms, while their spongy structure helps them absorb blood and exudates and provide compression properties (Figure 5B).^35^ Experimental studies conducted that the cryogel displayed excellent photothermal responsiveness as an antimicrobial agent in wounds and accelerated wound healing. The cryogel is also resistant to UV irradiation and could be used repeatedly for antimicrobial action under NIR light. Besides, in order to maintain the activity of growth‐promoting peptides and antimicrobial drugs, our group has proposed SF protein/gelatin hybrid particles with the inverse opal structure.^36^ Due to the sufficient mechanical strength, the Black phosphorus quantum dots (BPQDs)‐doped silk proteins were used as rigid scaffolds. Additionally, gelatin containing growth factors and antimicrobial peptides was used to fill nanopores inside SF inverse opals. Therefore, the drugs in the secondary hydrogels were well protected, overcoming the issues associated with direct administration of drugs in the microstructures of the SFIO scaffold. Through rapid conversion of light energy into heat, BPQDs increased local temperatures when exposed to NIR light, leading to melting of the external gelatin hydrogel, thus achieving a controlled release of both VEGFs and antibacterial peptides. Benefiting from these properties, these SF protein/gelatin hybrid particles exhibited great potential in wound healing.

### 4.2 Tumor treatment

**FIGURE 6:**
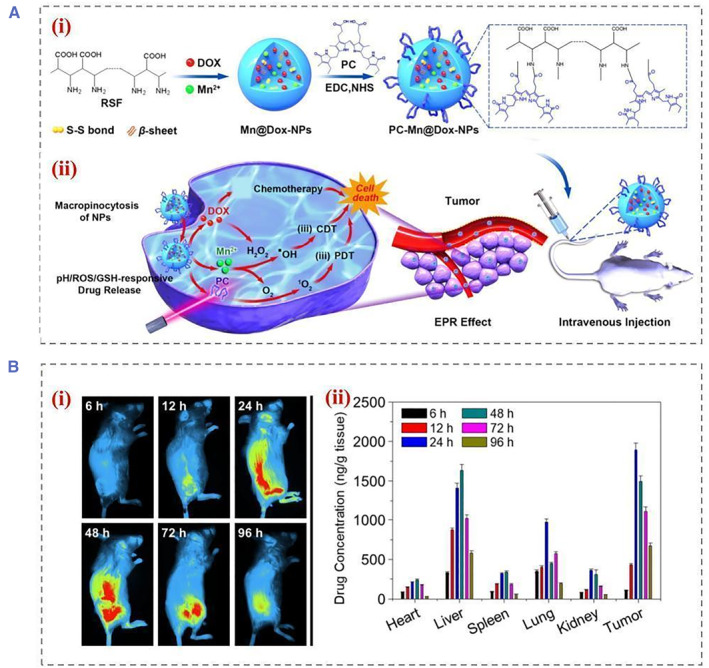
(A) Schematic diagram of (i) the construction of PC‐Mn@Dox‐NPs, (ii) schematic illustration of the cellular drug uptake process and the polyreactive drug release process, and the synergistic therapeutic strategy of PC‐Mn@Dox‐NPs for cancer treatment through cascade reactions. (B) The living images in vivo (i) for biodistribution and pharmacokinetic study of NPs, (ii) quantification of fluorescence intensities of tumors and organs after injection of NPs. Reproduced with permission.^38^ ^a^ Copyright 2021, American Chemical Society. NP, nanoparticle.

Surgical resection and radio ablation are the most common treatments for eradicating localized and non‐metastatic tumors. Chemotherapy is the only way to treat diseases that have spread throughout the body.^37^ A conventional chemotherapy agent affects both cancerous and normal cells randomly throughout the body. Consequently, the drug dose within the tumor cannot be delivered effectively due to this distribution formation, which results in poor therapeutic efficacy.^38^ Conventional small molecule drugs have a number of disadvantages, including off‐target effects and low bioavailability. In order to eliminate these drawbacks, controllable photodynamic therapy (PDT) via localized regions is a promising option. SF‐based multi‐responsive nanoparticles have been used for combined anti‐cancer therapy. For example, drugs were encapsulated in SF‐based nanoparticles and conjugated to surfaces with phycocyanin (PC) molecules (Figure 6).^38^ ^a^ Through enhanced permeability and retention (EPR) effect, the PC‐Mn@Dox‐NPs accumulated in mice's tumor areas after intravenous injection and were efficiently absorbed by the tumor cells. As a result, PC‐Mn@Dox‐NPs responded to the TME by accelerating the rate of drugs release in the tumor cells. DOX inhibited tumor cell viability as well as increased H_2_O_2_ production for tumor cells.

**FIGURE 7:**
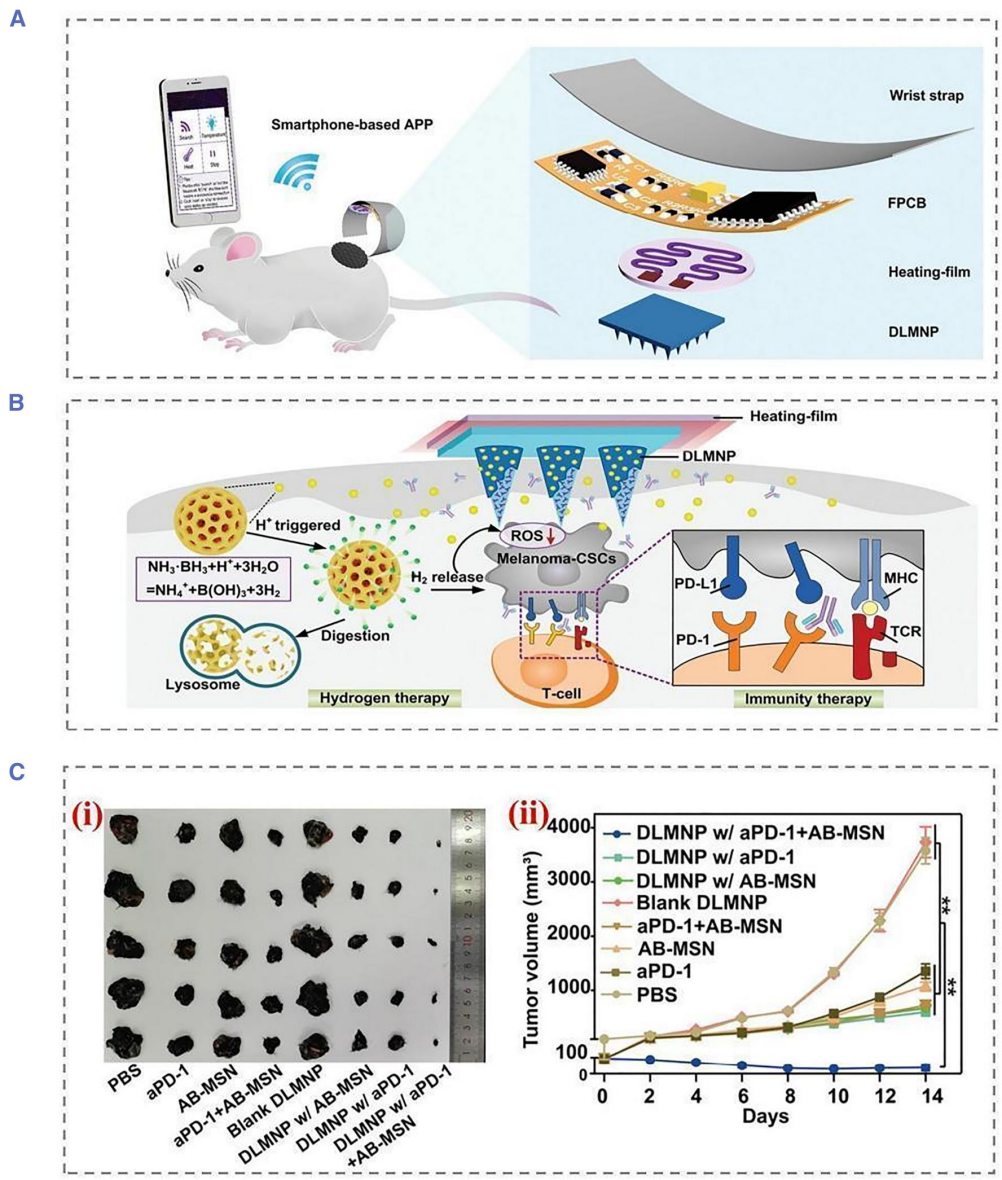
(A) Schematic showing wirelessly connected SMND percutaneous drug delivery to a mouse melanoma model. The SMND is mainly composed of a double‐layer MN patch (DLMNP), a heating film, a flexible printed circuit board (FPCB), a smartphone‐based application (APP), and a wristband. (B) Structure of the double‐drug‐containing DLMNP. SF loaded on PD‐1 is the inner matrix of DLMNPs, while PCL encapsulated by AB‐MSN is the outer thermally responsive coating of DLMNPs. (C) Representative photographs in (i) were tumors from treated mice, (ii) tumor growth curve of B16‐CSCs tumor‐bearing mice within 14 days of treatment. All significant differences in the figure are the results of comparison with the PBS group. Reproduced with permission.^39^ Copyright 2022, John Wiley and Sons. PBS, phosphate buffered saline.

On‐demand drug delivery devices can also be combined with smart devices. In immunotherapy and hydrogen therapy, a silk‐based microneedle device (SMND) has been developed for thermally responsive drug release (Figure 7).^39^ By combining thermally responsive MNs with an in vitro detection device, the SMND enabled controlled drug delivery, as well as on‐time monitoring.^40^ SF has excellent biocompatibility and tunable mechanical properties, making the SF hydrogel ideal for MNs. In addition, post‐treatment could be administered to the SF MN controlled dissolution, resulting in sustained transdermal drug release. When the MN was embedded in situ in the tumor and changed temperature with a heating coating, thermally converting polycaprolactone to a liquid state for the H_2_ release. In animal melanoma cancer stem cell (CSCs) models, MN‐mediated co‐immunotherapy and local delivery of hydrogen therapy significantly improved the efficacy of antitumor and anti‐CSCs therapies as well as decreased systemic toxicity and side effects. This study provided a unique strategy for the effective and safe management of life‐threatening cancers through synergistic immunotherapy and hydrogen therapy.

### 4.3 Immunomodulatory

**FIGURE 8:**
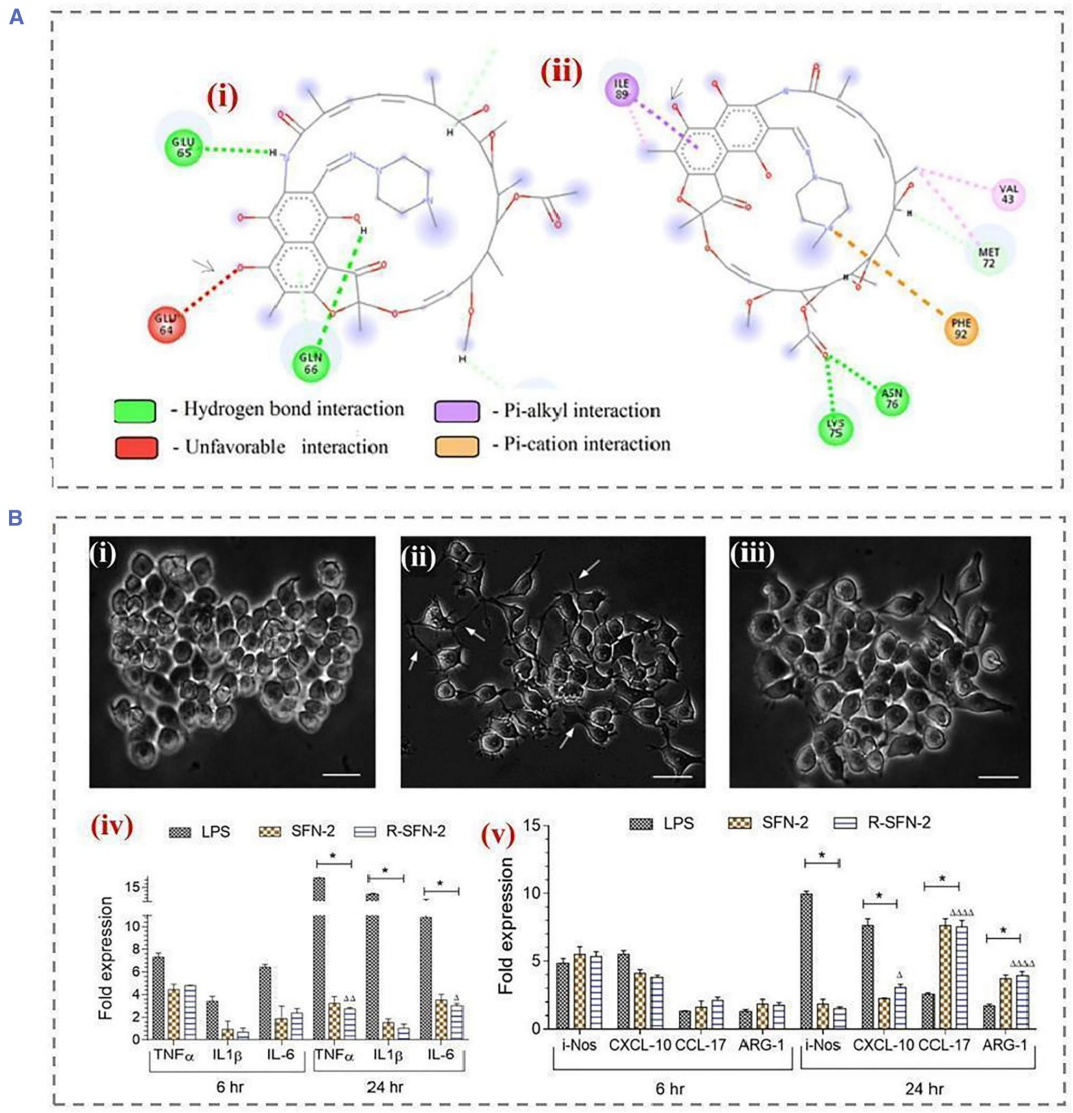
(A) Interaction of (i) RIF with silk fibroin at pH 7.2; arrows indicate unfavorable electrostatic interactions between the 4‐hydroxyl group of rifampicin and GLU64 of silk fibroin (red). (ii) RIF and silk fibroin at pH interactions at 3.8; arrows indicate the formation of favorable interactions. (B) Effects of pH‐induced self‐assembled silk fibroin nanoparticles (SFN‐2, 100 μg/mL) on the morphology of RAW 264.7 cells observed by phase contrast microscopy after 24 h. (i) Untreated naive macrophages, (ii) lipopolysaccharide‐treated macrophages, and (iii) R‐SFN‐2‐treated macrophages. (iv) Proinflammatory cytokine expression profile of RAW264.7 macrophages when exposed to SFN‐2. (v) Anti‐inflammatory cytokine expression profiles upon exposure to SFN2. Reproduced with permission.^44^ Copyright 2022, American Chemical Society.

SF nanoparticles generated from SF have endogenous anti‐inflammatory activity and mucosal healing properties. The immune response is a key parameter in assessing biocompatibility. Reprogramming cells by means of genetic engineering and stem cells can be used for specific therapeutic purposes.^41^ However, these approaches face challenges regarding of vector immunogenicity and in vivo delivery efficiency.^42^ Recently, alternative techniques for induced reprogramming cells using biomaterials or small molecules independent of transcription factors have been used to overcome these limitations. Current studies suggest that SF is a kind of immunogenic due to the presence of a hydrophobic *β*‐sheet structural domain. Hydrophobic surfaces have been reported to be more likely to induce chronic inflammation because of the high affinity of adsorbed proteins for hydrophobic surfaces. The hydrophobic region of SF exposes binding sites to platelets, neutrophils, and other phagocytes, which can adhere and activate the secretion of pro‐inflammatory cytokines to recruit different immune cells. This leads to chronic inflammation and further causes macrophage polarization.^43^ However, it has been shown that macrophages are able to polarize toward anti‐inflammatory (M2) type, and this type of immune response is beneficial for a variety of applications.^44^ In particular, it was also reported that following exposure to SF nanoparticles, macrophages became M1 inflammatory within 6 h and M2 anti‐inflammatory within 24 h (Figure 8). Additionally, SF modulates the inflammatory response by reducing Il‐18, Il‐1β, iNOS, and COX‐2, which encodes pro‐inflammatory mediators.

**FIGURE 9:**
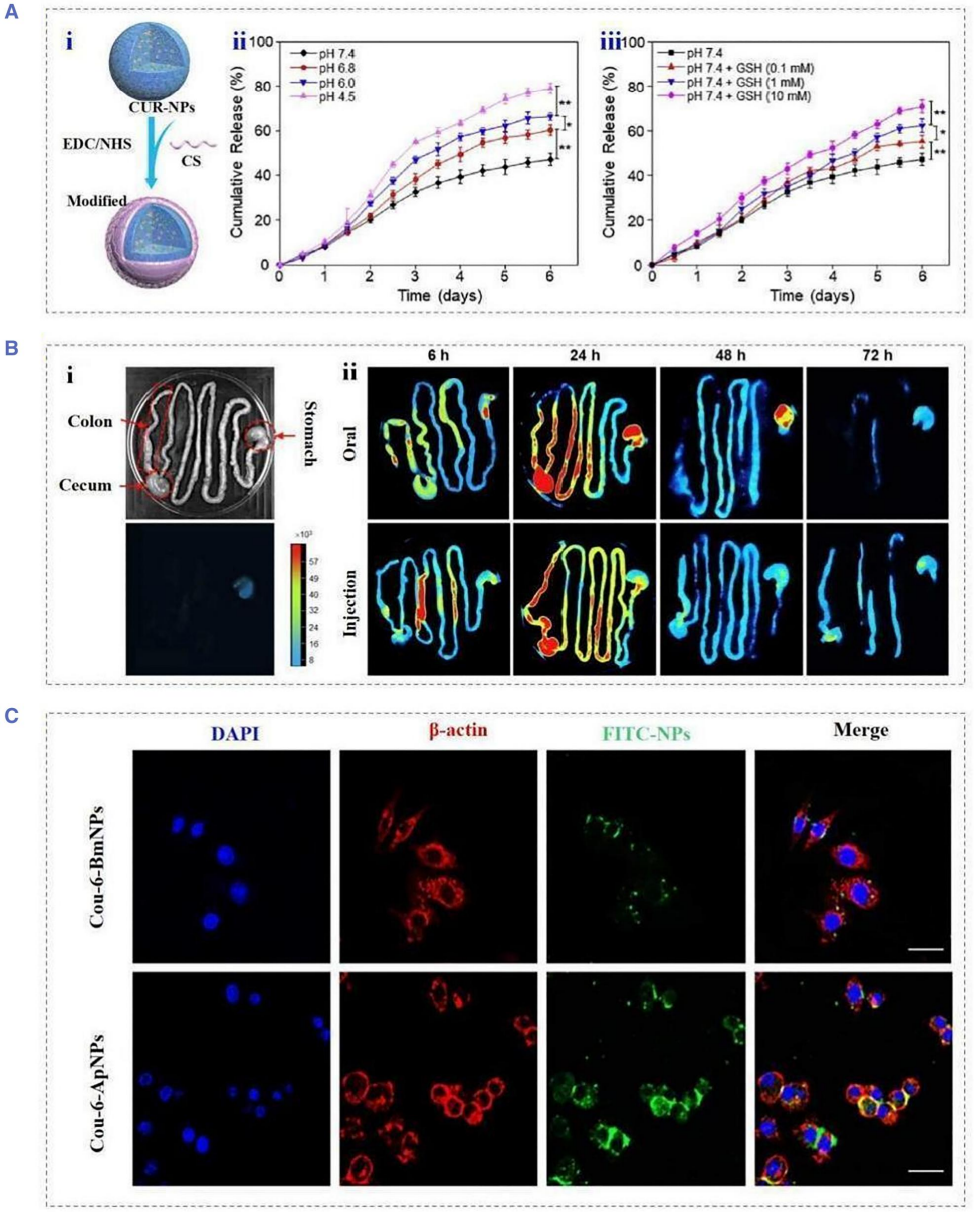
(A) Schematic illustration of (i) the preparation process of functionalized silk fibroin nanoparticles, (ii) in vitro cumulative drug release profiles in buffer solutions with different pH values and (iii) GSH concentrations. Reproduced with permission.^5^ ^b^ Copyright 2019, Elsevier. (B) Brightfield and fluorescence images of (i) the GIT of the control group (untreated with NP). (ii) Representative images of GIT showing biodistribution of CS‐Cy7‐NPs administered orally or intravenously at different time points (6, 24, 48, and 72 h). (C) Detection of Cou‐6‐BmNPs and Cou‐6‐ApNPs in CT‐26 cells in the presence of RGD molecules after 2 h incubation ApNPs. Reproduced with permission.^46^ Copyright 2022, Elsevier.

SF nanoparticles enable responsive drug delivery by responding to the endogenous microenvironment. Repairing the gut of patients with inflammatory bowel disease requires both anti‐inflammatory and mucosal tissue repair. Benefiting from the inherent anti‐inflammatory properties of SF, responsive SF nanoparticles can be used as responsive carriers for the treatment of inflammatory bowel disease.^45^ SF nanoparticles loaded with anti‐inflammatory drugs were studied for the treatment of ulcerative colitis (Figure 9A).^5^ ^b^ The surface of SF is modified with chondroitin sulfate to improve the uptake of nanoparticles by cells. It was also proved that the generated nanoparticles can be used in the treatment of ulcerative colitis. Endogenous responsive drug release was achieved in a simulated microenvironment in the presence of lysosomes, glutathione, and ROS. The surface of *Antheraea pernyi* SF is rich in the R‐G‐D sequence. In another attempt, to enhance the bioavailability of responsive SF nanoparticles, Ma et al. believed that there were a large number of receptors that specifically bind to the RGD sequence in the inflamed colon tissue cells (Figure 9C).^46^ After encapsulating fluorescent probes in *Antheraea pernyi* SF nanoparticles, it was demonstrated that tussah fibroin nanoparticles can be taken up by inflammatory tissue cells through RGD‐mediated endocytosis. In vitro drug release experiments showed that the *Antheraea pernyi* SF has similar multi‐responsiveness to bombyx mori SF. Further, the biodistribution of Cy7‐labeled nanoparticles in mice was tracked by in vivo imaging, and the *Antheraea pernyi* SF group had more fluorescence intensity in the colon after 72 h.

### 4.4 Others

The SF solution takes a lot of time to form a hydrogel in its natural state by self‐assembly, mainly through the transformation of free polypeptide fragments into a Silk II structure under extremely stringent conditions. Liu et al. introduced a surfactant that induces SF self‐assembly at body temperature.^47^ It was showed that the unique pathway and thermodynamics of forming fast gels: fast spreading and more formation of Silk II structures are crucial factors for ultrafast SF self‐assembly. They induced ultrafast gelation of SF by mixing a biocompatible amino acid surfactant, and an injectable antibacterial and biodegradable hemostatic hydrogel was prepared using this temperature‐sensitive ultrafast gel induced by ethyl lauroyl arginine hydrochloride (LAE) for the treatment of incompressible hepatic hemorrhage. The surfactants could also induce changes in the conductivity and solubility of bacterial membranes, thereby inhibiting bacterial growth. In addition, the positively charged guanidine groups on LAE can strongly interact with negatively charged bacterial membranes through electrostatic attraction, thereby achieving an effective bactericidal effect after thermally induced gelation. The employment of scaffolds to deliver electrical stimulation in clinical is still challenging. Recently, Yang et al. proposed a novel biocompatible SF scaffold controlled by photoacoustics, which can stimulate nerve tissue to release nerve growth factor as a drug to promote the regeneration and growth of nerve cells by photoacoustic stimulation.^48^ Embedded carbon nanotubes (CNTs) absorb pulsed NIR‐II light and convert light energy into sound waves, which activate neurons cultured on CNT/silk. Compared with the reported work on the stimulation of nanoparticles under NIR excitation, the scaffold approach avoids the difficulty of controlling the position of injected nanoparticles.

## 5 SUMMARY AND OUTLOOK

Stimulus‐responsive materials are an emerging class of materials for multifunctional applications. The inherent processability, thermal stability, and mechanical strength of SF make SF stand out for on‐demand drug delivery. Considering recent advances in the field of nanomaterials, further integration of various responsive microcarriers into SF‐based smart delivery systems is bound to bright the future of on‐demand drug release.^49^ In this review, we discuss several techniques about the preparation of controlled release carriers made from SF, including a description of the properties, secondary structure and gelling mode of silk proteins. The water solubility allows for the manufacture of highly tunable forms under mild conditions. Simple methods such as adjusting the release curve by changing its concentration, increasing the multiple reaction mode or changing the beta‐fold content are tools to make SF particularly attractive. In addition, simple surface modifications prevent the binding and release process of the material in transit, thus enabling targeted delivery. Taking advantage of these features, powerful on‐demand drug delivery techniques based on SF have been created. These methods use both exogenous and endogenous stimuli, including ultrasound, electromagnetic fields, pH responses, etc., to trigger on‐demand drug release. Depending on the material and design of the drug carrier, SF‐based drug delivery systems could be applied to wound healing, tumor ablation, and immune modulation.

Although these advantages of SF have led to its development for on‐demand drug delivery, there remain many challenges. As a natural material, SF may have different properties due to species differences and extraction processes. Besides, residues of silk glue during degumming may lead to uncertain immunogenicity, making it hard for the quality control of SF delivery systems as well as prediction of its release kinetics. Handful current studies have attempted to develop multi‐responsive drug carriers. These are critical because there are often changes in the target microenvironment from several different environmental factors that can occur simultaneously, making a single stimulus insufficiently responsive. In addition, future delivery tools will be tailored to different patient characteristics. We believe that SF‐based on‐demand drug delivery platforms will provide safe, stable, cost‐effective treatments in the near future to address medical needs that still unmet in clinical care.

## AUTHOR CONTRIBUTION

Yuanjin Zhao conceived the idea; Xiang Lin and Lijun Cai wrote the manuscript and edited the figures; Xinyue Cao revised the manuscript; Yuanjin Zhao supervised the manuscript.

## CONFLICT OF INTEREST STATEMENT

The authors declare no conflict of interest.
